# Predictors and patterns of suicidal ideation disclosures among American adults

**DOI:** 10.1111/sltb.13126

**Published:** 2024-09-02

**Authors:** Taylor R. Rodriguez, Shelby L. Bandel, Samantha E. Daruwala, Michael D. Anestis, Joye C. Anestis

**Affiliations:** ^1^ The New Jersey Gun Violence Research Center Rutgers University Piscataway New Jersy USA; ^2^ Department of Psychology Rutgers University Piscataway New Jersy USA; ^3^ The Ohio State University Wexner Medical Center Columbus Ohio USA; ^4^ Department of Urban‐Global Public Health, School of Public Health Rutgers University Piscataway New Jersy USA; ^5^ Department of Health Behavior, Society, and Policy, School of Public Health Rutgers University Piscataway New Jersy USA

**Keywords:** suicidal thoughts, suicide disclosures, US adults

## Abstract

**Introduction:**

When experiencing suicidal thoughts, many individuals do not tell others, making it difficult to ensure suicide prevention resources reach those who need it.

**Methods:**

The current study utilizes a large sample of US adults who have experienced suicidal ideation in their lifetime (*n* = 1074) to examine predictors of disclosures. We also explore who participants disclose to and how helpful these disclosures are rated.

**Results:**

A majority (*n* = 812, 75.6%) reported disclosing. Black and Hispanic participants were less likely to disclose than White participants. Those who were never married were more likely to disclose, as were those who have attempted suicide. Mental healthcare utilization and favorable attitudes toward mental healthcare were also positive predictors of disclosure. More participants reported disclosing to a personal connection (*n* = 532, 65.5%) than a mental health professional (*n* = 282, 34.8%). On average, most sources were rated as neither helpful nor harmful.

**Conclusion:**

The study highlights those who may be at a higher risk of experiencing suicidal thoughts but going unidentified. To increase helpfulness of disclosures, suicide prevention programming should emphasize training for laypersons and professionals on how to effectively respond when someone reveals that they are thinking of suicide.

## INTRODUCTION

Suicide is a significant public health concern in the United States, and the age‐adjusted suicide rate has been increasing since 2001 (Garnett & Curtin, 2023). Despite a decline in 2019 and 2020 (Garnett & Curtin, 2023), suicide rates have continued to increase, with provisional data indicating that the rate reached an all‐time high in 2022 (Curtin et al., 2023). Suicide prevention initiatives are a national priority and have expanded over the years (US Surgeon General, 2021). Yet, many individuals do not disclose suicidal thoughts, which can hamper efforts to detect and mitigate suicide risk (Cukrowicz et al., 2014). Rates of disclosure of suicidal thoughts and behaviors can widely vary (Hallford et al., 2023), with one study finding a rate as low as 8% in a clinical sample (Osvath et al., 2003) and others reporting rates around 50% in community samples (e.g., Cukrowicz et al., 2014; Husky et al., 2016). Better understanding of the factors associated with disclosure and non‐disclosure of suicidal thoughts and/or behaviors is needed to further inform suicide prevention efforts that can reach subgroups of individuals who are less likely to disclose.

Research examining sociodemographic and psychological correlates of disclosure have found mixed results. For example, studies have found mixed findings for age and gender; some studies identified that younger age, older age, and male gender are correlated with lower rates of disclosure, while others have found no significant association between these factors (e.g., Cukrowicz et al., 2014; Eskin, 2003; Husky et al., 2016). Lower rates of disclosure have also been associated with race/ethnicity. Black and Hispanic individuals have been less likely to report past‐year suicidal ideation but more likely to report a suicide attempt compared to White individuals (Bommersbach et al., 2023). Due to differences in expressions of distress and structural racism, these minoritized groups may not disclose thoughts, while behaviors may be perceived as more severe and warranting disclosure (Bommersbach et al., 2023). Racially minoritized adolescents, compared to White peers, disclosed suicidal ideation less often, citing fear of negative reactions and resistance to intervention as main reasons to withhold their experiences (Shin et al., 2023). Ultimately, racially minoritized groups seem to disclose less often, while there are still some questions regarding disclosures based on age and sex.

In addition to individual or demographic variables, interpersonal and mental health variables are important when considering whether individuals disclose their suicidal thoughts (Fulginiti et al., 2016). Individuals are less likely to disclose when they have less social support and connectedness, higher levels of stigma (Fulginiti et al., 2016), and lower quality of life (Cukrowicz et al., 2014). Disclosures may, in part, depend on the availability of trustworthy sources to talk to, as well as more openness to mental healthcare/less stigma. On the other hand, greater likelihood of disclosure is associated with increased suicidal behavior (Chu et al., 2011; Encrenaz et al., 2012; Husky et al., 2016) and history of mental health treatment (Cukrowicz et al., 2014). Similar to the race‐based findings previously cited (i.e., Bommersbach et al., 2023), it is likely that increased severity of suicidal experiences (i.e., behaviors rather than ideation alone) may prompt individuals to seek support. This may also prompt professional help‐seeking and having access to a provider in treatment can allow for more opportunity to disclose.

Calear and Batterham (2019) used a large sample of Australian adults to examine several correlates of disclosure to specific sources, the perceived helpfulness of the source after the disclosure, and the number of disclosure sources. Disclosure of suicidal ideation to a range of sources (e.g., friend, parent, and/or psychologist, etc.) and the total number of disclosures was associated with younger age (i.e., 18–35 years), more severe suicidal ideation, a suicide attempt in the past 12 months, and lower levels of suicide stigma. Several participant characteristics were associated with perceived helpfulness of the source following disclosure. The response of a general practitioner was perceived as less helpful after a disclosure among individuals who were younger, spoke languages other than English, and reported greater severity of ideation and personal stigma. Disclosure to phone helplines were less likely to be perceived as helpful among individuals reporting a suicide attempt in the past 12 months; however, helplines were rated as more helpful when individuals reported greater severity of ideation. While this study offers an important contribution, replication in a U.S. sample is needed to determine generalizability.

Hallford et al. (2023) recently conducted a systematic review and meta‐analysis to estimate the prevalence of disclosure of suicidal thoughts and behaviors and to identify which subgroups individuals disclose to with studies primarily in the United States or European countries. They examined several sociodemographic (i.e., age, percentage of females) and psychological factors (i.e., lifetime suicide attempt, percentage of the sample with current psychiatric disorder) as moderators of disclosure rates. They estimated that less than 50% of individuals had disclosed suicidal ideation or behaviors and found higher prevalence of disclosure was associated with female gender, higher prevalence of psychiatric disorders, and a longer timeframe of suicidal thoughts or behaviors. Prevalence of disclosure was lower when it was reported verbally rather than written online and when the source was samples of suicide decedents compared to community samples. In terms of disclosure sources, Hallford et al. (2023) reported that the prevalence of disclosure and the number of disclosures were numerically higher for family members than for friends or professionals; however, they were unable to directly compare the proportions across the subgroups. While their findings provide a clearer estimate of the prevalence of disclosure rates and to whom individuals may prefer to disclose, the authors note that more research is needed on factors that impact disclosures and studies with community samples should expand beyond convenience sampling methods.

The current study sought to examine several factors that may impact whether American adults disclose their suicidal ideation. As a partial replication of Calear and Batterham's (2019) study focused on Australian adults, we examined the following sociodemographic predictors: age, race/ethnicity, biological sex, sexual orientation, education level, income, location of residence (i.e., rurality), marital status, and suicide attempt history. Rather than looking at these factors in isolation, we also included variables associated with suicide risk and mental health. These include firearm ownership status, past mental health service utilization, attitudes toward professional mental health help seeking, and mistrust toward others as potential predictors of lifetime suicidal ideation disclosure. We were particularly interested in examining firearm ownership because firearms are highly lethal (Conner et al., 2019) and are the leading method for suicide in the United States (Stone et al., 2021). Recent research suggests that individuals who die by firearm suicide may be less likely to seek mental health treatment (Bond et al., 2022). While the included sociodemographic and suicide‐related variables are relevant in current literature, patterns of suicide disclosures seem to vary. As such, our analyses are exploratory in nature.

Our second aim is to explore who individuals tend to disclose their suicidal ideation to and how helpful they rate these disclosures. The sources included in this study include personal connections (friends, intimate partners, parents, and other relatives), mental health professionals (psychiatrists, psychologists, social workers, counselors, and internet therapy programs), and other sources (doctors/general practitioners and suicide prevention hotlines). We expect that, overall, participants will report disclosing to personal connections more than professional sources. We also anticipate that disclosures to professionals will be rated as more helpful than disclosures to personal connections.

## METHODS

### Procedure and participants

The present study utilized a subset of data from a larger data collection derived from KnowledgePanel (KP) via Ipsos from April 29 to May 15, 2022. KP is a probability‐based panel designed to be representative of the general population of the United States. All procedures were approved by the Rutgers Biomedical and Health Services Institutional Review Board and all participants provided informed consent prior to participation. Data collection focused on five states across the United States: New Jersey, Mississippi, Minnesota, Texas, and Colorado. The entire sample included 3510 participants and data were weighted for geodemographic distribution based on benchmarks from the 2019 American Community Survey. This study utilized data weighs relative to the full sample rather than participants' state of residence. Furthermore, the present study isolated the sample to only those who endorsed suicidal ideation in their lifetime (*n* = 1074) on the self‐injurious thoughts and behaviors interview‐short form‐self report (SITBI‐SF‐SR; Nock et al., 2007).

See Table 1 for all sample demographics. The average age of the sample was 41.26 (SD = 15.46, range 18–94), and most participants were female (*n* = 619, 57.6%). Regarding race/ethnicity, most participants (*n* = 571, 53.2%) identified as White while 308 (28.7%) identified as Hispanic, and 118 (11.0%) identified as Black. A majority of the sample were heterosexual (*n* = 861, 80.1%), currently married (*n* = 464, 43.2%), and living in non‐metropolitan rural areas (*n* = 437, 40.7%). Considering highest educational level attained, most participants either attended some college/obtained an associate degree (*n* = 256, 33.1%) or obtained a high school diploma or GED (*n* = 278, 25.9%). Most participants reported a household income between $25,000–$49,999 (*n* = 234, 21.8%) or $50,000–$74,999 (*n* = 220, 20.55).

**TABLE 1:** Demographic breakdown of the sample.

Variables	*n* (%)
Biological sex	
Male	455 (42.4%)
Female	619 (57.6%)
Race/Ethnicity	
White	571 (53.2%)
Hispanic	308 (28.7%)
Black	118 (11.0%)
Other	63 (5.9)
Multiracial (2+)	14 (1.3%)
Sexual orientation	
Heterosexual	861 (80.1%)
Gay or Lesbian	46 (4.3%)
Bisexual	91 (8.5%)
Pansexual	12 (1.1%)
Asexual	16 (1.5%)
Other	24 (2.2%)
Chose not to disclose	23 (2.2%)
Education level	
No HS diploma/GED	121 (11.3%)
HS graduate/GED	278 (25.9%)
Some college/Associate's	356 (33.1%)
Bachelor's degree	190 (17.7%)
Graduate degree	130 (12.1%)
Household income	
< $10,000	69 (6.4%)
$10,000–$24,999	95 (8.9%)
$25,000–$49,999	234 (21.8%)
$50,000–$74,999	220 (20.5)
$75,000–$99,999	119 (11.1%)
$100,000–$149,999	183 (17.0%)
> $150,000	155 (14.4%)
Marital status	
Currently married	464 (43.2%)
Never married	445 (41.4%)
Other: widowed, divorced, separated	166 (15.5%)
Rurality	
Non‐metropolitan rural	437 (40.7%)
Metropolitan rural	275 (25.6%)
Urban	360 (33.5%)

A minority of the sample owned a firearm (*n* = 258, 24.0%). Additionally, 120 participants (11.1%) reported attempting suicide in their lifetime. A total of 434 participants (40.4%) utilized services in their lifetime, and 371 (34.5%) were reportedly in services at the time of the survey.

### Measures

#### Self‐Injurious Thoughts and Behaviors Interview‐Short Form‐Self Report (SITBI‐SF‐SR; Nock et al., 2007)

The SITBI‐SF‐SF is a self‐report questionnaire regarding suicidal thoughts and behaviors. The present study utilized a subset of questions regarding lifetime suicidal thoughts and attempts. Inclusion criteria involved endorsing one of the included suicidal thoughts within the SITBI‐SF‐SR (e.g., “Maybe I should kill myself.”). The suicide attempt item asked if participants “tried to kill” themselves in their lifetime.

#### Sociodemographic information

De‐identified sociodemographic information was collected via KP user profiles and provided to the research team. Self‐reported demographics include biological sex, race/ethnicity, age, education, household income, sexual orientation, marital status, and rurality. Rurality was calculated using ZIP code data, converted to population density, and classified as “non‐metropolitan rural,” “metropolitan rural,” and “urban.” Race/ethnicity categories (participants selected one) included White/Non‐Hispanic, Black/Non‐Hispanic, multi‐racial/non‐Hispanic or other, and Hispanic.

Internally developed questions also inquired about firearm ownership (yes or no to current ownership) and mental healthcare utilization. Participants were asked if they have utilized individual or group therapy, prescription medication, psychological assessment/testing, or other services for mental health difficulties. Lifetime utilization was coded as yes if they endorsed utilizing any of these services.

#### Suicidal ideation disclosures

Participants were asked “Please indicate which of the following individuals—if any—you have previously told about your thoughts of suicide.” Response options included: intimate partner (e.g., boyfriend, wife), parent, friend (not related), other relative/family member, psychiatrist, psychologist, social worker, internet therapy program, counselor, doctor/general practitioner, and suicide prevention hotline (e.g., National Suicide Prevention Lifeline). Based on their responses, participants could then rate how helpful or harmful the disclosure was on a scale from 1, “Extremely Harmful” to 5, “Extremely Helpful.”

#### Attitudes Toward Seeking Professional Psychological Help Scale‐Short Form (ATSPPH‐SF; Fischer & Farina, 1995)

The ATSPPH‐SF is a 10‐item measure with a 4‐point Likert‐type response option (0 = disagree, 4 = agree). Higher summed scores were indicative of more openness to seeking treatment and greater perceived value of treatment (Elhai et al., 2008). The measure has demonstrated good internal consistency with coefficient alphas ranging from 0.77 to 0.84 in prior research (see Elhai et al., 2008, for a brief review). Internal consistency reliability for the scale was acceptable (*α* = 0.85).

#### Modified‐Posttraumatic Cognitions Inventory (PTCI; Foa et al., 1999)

The PTCI includes items focused on negative cognitions about self, negative cognitions about the world, and self‐blame. Three of the PTCI items regarding negative cognitions about the world were presented, without the original instructions which focus on traumatic experiences, to allow for a broader look at individuals' distrust or sensitivity to threats. These include: “People can't be trusted,” “People are not what they seem,” and “I can't rely on other people.” Item responses range from 1, “Totally Disagree” to 7, “Totally Agree.” Although the PTCI includes more items than the present study, these items have been used in isolation in prior work (e.g., Anestis & Bryan, 2021). Internal consistency reliability for the included items was acceptable (*α* = 0.85), supporting the use of total scale scores for the three items.

### Data analytic plan

To examine Aim 1 and identify sociodemographic predictors of suicidal ideation disclosure, a hierarchical logistic regression was utilized to identify those who disclosed their suicidal ideation in their lifetime (*n* = 812, 75.6%) and those who have never told anyone about their suicidal ideation (*n* = 250, 23.2%). The first step of the regression included all demographic variables: age, sex, race/ethnicity, sexual orientation, highest education attained, household income, marital status, and rurality. The second step included variables related to suicide risk including a history of suicide attempts and firearm ownership status. Predictors related to mental health were entered into the third step: mental healthcare treatment utilization in lifetime, attitudes toward seeking professional mental healthcare, and trust toward others. The sources of suicidal ideation disclosures and their level of helpfulness were explored (Aim 2) with descriptive analyses. A *t*‐test indicated whether helpfulness ratings differed across professional and personal sources.

## RESULTS

Zero order correlation coefficients and multicollinearity statistics (see Table 2) were obtained for all study variables. No issues with multicollinearity were evident, as all variance inflation factors (VIFs) were below 5, tolerance values were above 0.2, and correlation coefficients were below 0.50 (Cohen, 1977; Kim, 2019).

**TABLE 2:** Intercorrelations and multicollinearity statistics for all study variables.

Study variables	1	2	3	4	5	6	7	8	9	10	11	12	13	14	VIF	Tolerance
Dependent variable																
Disclosed suicidal ideation (1)	‐	−0.07	0.11	−0.08	0.09	0.04	0.04	0.07	0.01	0.10	−0.06	0.16	0.23	−0.03		
Demographic variables																
Age (2)		‐	0.01	−0.24	−0.25	0.06	0.15	−0.48	−0.02	−0.04	0.16	0.01	0.01	−0.11	1.16	0.87
Biological sex (3)			‐	−0.06	0.09	−0.02	−0.05	−0.05	0.01	0.05	−0.11	0.05	0.21	0.08	1.01	0.93
Race/Ethnicity (4)				‐	−0.10	−0.08	−0.16	0.18	0.15	0.01	−0.14	−0.14	−0.07	0.13	1.15	0.87
Sexual orientation (5)					‐	−0.05	−0.07	0.19	0.00	0.10	−0.15	0.01	0.14	0.08	1.13	0.88
Education attained (6)						‐	0.51	−0.12	0.17	−0.12	0.03	0.06	0.23	−0.20	1.46	0.68
Household income (7)							‐	−0.29	0.01	−0.11	0.16	0.05	0.11	−0.20	1.52	0.66
Marital status (8)								‐	0.10	0.03	−0.22	0.04	−0.02	0.04	1.10	0.91
Rurality (9)									‐	0.01	−0.11	0.04	0.03	0.00	1.08	0.93
Suicide‐related variables																
Suicide attempts, lifetime (10)										‐	−0.04	0.22	0.07	0.04	1.08	0.92
Firearm owner (11)											‐	−0.04	−0.14	0.05	1.13	0.89
Mental health‐related variables																
Service utilization, lifetime (12)												‐	0.27	−0.02	1.17	0.86
Attitudes toward help‐seeking (13)													‐	−0.29	1.4	0.71
Trust toward others (14)														‐	1.25	0.80

*Note*: Scores above 5 indicate high multicollinearity. Tolerance scores below 0.2 indicate high multicollinearity. Correlation coefficients between independent variables above 0.5 indicate a large association and issues with multicollinearity.

Abbreviation: VIF, variance inflation factors.

### Who is more likely to disclose their suicidal ideation to others?

The regression model (Table 3) accounted for 16% of the variance in whether participants disclosed their suicidal ideation; 8% was accounted for by the first step including demographic variables, this increased by 1% when the suicide‐related variables were added, and 5% more with the mental health‐related variables. Race/ethnicity emerged as a significant predictor of suicidal ideation disclosures. Compared to White participants, Black (OR = 0.28, 95% CI [0.17–0.46]), and Hispanic (OR = 0.66, 95% CI [0.44–0.97]) participants were less likely to disclose their ideation to others. Those who have never been married were more likely to have disclosed their suicidal ideation compared to those who are currently married (OR = 1.69, 95% CI [1.12–2.55]). As expected, those who have attempted suicide were almost twice as likely to disclose their ideation compared to those who have never attempted (OR = 1.95, 95% CI [1.03–3.71]). Unexpectedly, trust toward others did not emerge as a significant predictor; however, the other mental health‐related variables did. Those who have utilized mental healthcare (OR = 1.52, 95% CI [1.09–2.12]) and with more positive attitudes toward professional mental healthcare services (OR = 1.08, 95% CI [1.05–1.11]) were more likely to disclose their suicidal ideation.

**TABLE 3:** Hierarchical logistic regression predicting whether participants disclosed suicidal ideation.

Study variables	*B* (SE)	Wald	*p*	Odds ratio	95% CI	*R* ^2^
Demographic variables						0.08
Age	−0.01 (0.01)	1.77	0.18	0.99	0.98–1.00	
Biological sex	0.27 (0.17)	2.63	0.11	1.31	0.95–1.81	
Black	−1.27 (0.25)	25.03	<0.001*	0.28	0.17–0.46	
Hispanic	−0.42 (0.20)	4.37	0.04*	0.66	0.44–0.97	
Other racial/ethnic identity	−0.22 (0.33)	0.42	0.51	0.81	0.42–1.55	
Sexual minority	0.19 (0.25)	0.56	0.45	1.20	0.74–1.95	
Education attained	−0.05 (0.09)	0.38	0.54	0.95	0.80–1.13	
Household income	0.02 (0.06)	0.15	0.70	1.02	0.91–1.15	
Never married	0.52 (0.21)	6.12	0.01*	1.69	1.12–2.55	
Widowed, divorced, separated	0.03 (0.24)	0.01	0.91	1.03	0.64–1.64	
Metropolitan rural	−0.26 (0.20)	1.60	0.21	0.78	0.52–1.15	
Urban	0.04 (0.20)	0.05	0.83	1.04	0.71–1.54	
Suicide‐related variables						0.09
Suicide attempts, lifetime	0.67 (0.33)	4.18	0.04*	1.95	1.03–3.71	
Firearm owner	−0.04 (0.19)	0.04	0.84	0.96	0.66–1.41	
Mental health‐related variables						0.16
Service utilization, lifetime	0.42 (0.17)	6.14	0.01*	1.52	1.09–2.12	
Attitudes toward help‐seeking	−0.08 (0.01)	28.01	<0.001*	1.08	1.05–1.11	
Trust toward others	−0.01 (0.02)	0.10	0.75	0.99	0.95–1.04	

Abbreviation: CI, confidence interval.

* < 0.05.

### Who do participants disclose to and which disclosure sources are most helpful?

We investigated the source of disclosures (Table 4) among those who report telling someone about their suicidal ideation (*n* = 812). Of note, participants were able to select all sources to whom they have disclosed and then rate the helpfulness of each source. As such, these groups are not exclusive. Most reported disclosing to one (*n* = 220, 27.1%) or two (*n* = 182, 22.4%) sources, while few reported disclosing to four or more sources (*n* = 135, 16.6%). More participants reported telling a personal connection (*n* = 532, 65.5%) than a mental health professional (*n* = 282, 34.8%). The source that was reported most often was an intimate partner (*n* = 325, 40.0%). The least endorsed source was an internet therapy program (*n* = 10, 1.3%); this source was considered within the mental health professional category.

**TABLE 4:** Descriptive analyses to identify disclosure sources and harmful/helpful ratings among.

Disclosure source^a^	Yes, disclosed thoughts^b^	Harmful/helpful rating^c^
	*n* (%)	Mean (SD)
Personal connection	532 (65.5%)	3.74 (0.94)
Intimate partner	325 (40.0%)	3.83 (1.04)
Friend	277 (34.1%)	3.78 (0.98)
Parent	171 (21.1%)	3.52 (1.25)
Other relative	147 (18.1%)	3.67 (0.91)
Mental health professional	282 (34.8%)	4.01 (1.04)
Psychiatrist	143 (17.6%)	3.99 (1.08)
Psychologist	132 (16.3%)	4.31 (0.88)
Counselor	108 (13.3%)	3.92 (1.12)
Social worker	45 (5.6%)	3.79 (0.84)
Internet therapy program	10 (1.3%)	3.59 (0.65)
Other		
Doctor/general practitioner	84 (10.3%)	3.88 (1.10)
Suicide prevention hotline	45 (5.5%)	3.73 (1.13)

^a^ Indiviudals were able to choose all sources they have told their suicidal thoughts. As such, these categories are overlapping.

^b^ Reflects percentage of the subsample of those who have disclosed (*n* = 812).

^c^ Participants rated responses on a scale of 1 (very harmful) to 5 (very helpful).

Given the low rates of disclosure within the other sources, doctors/general practitioners (*n* = 84, 10.3%) and the suicide prevention hotline (*n* = 45, 5.5%), our investigation of harmful/helpful ratings focused on personal connections and mental health professionals (Table 4). On average, most sources were rated as neither particularly helpful nor harmful (*M*_range_ = 3.52–4.31). Professionals were rated over a quarter point more helpful (M = 4.01, SD = 1.04) than personal connections (M = 3.74, SD = 0.94). The most highly rated source was psychologist with a mean helpful rating of 4.31 out of 5 (SD = 0.88). The lowest rated source was a parent with a mean helpful rating of 3.52 (SD = 1.25). A total of 210 participants (24.9% of those who disclosed [*n* = 812]) disclosed to both professional and personal connections. Among those who rated both, professionals (*M* = 4.03, SD = 1.06) and personal connections (*M* = 3.92, SD = 0.90) were deemed equally helpful (*t*(209) = 1.74, *p* = 0.084).

## DISCUSSION

This paper sought to expand upon prior literature by using a sample of U.S. adults to examine demographic and clinical factors associated with disclosing suicidal thoughts. Results indicated that those who identified as Black or Hispanic were less likely to disclose their suicidal thoughts relative to their White counterparts. This finding was not surprising, as minoritized populations face extensive barriers to mental health treatment due to longstanding structural racism. Such experiences may reduce individuals' opportunities to disclose their suicidal thoughts to mental health providers. Additionally, prior research has found that both Hispanic and Black Americans report greater mental health stigma relative to White Americans (DeFreitas et al., 2018; Uebelacker et al., 2012; Ward et al., 2013). Stigma may produce a barrier to disclosing these thoughts to personal connections as well. Given the growing suicide rate among minoritized populations, the present findings also suggest that more needs to be done to ensure these individuals feel comfortable disclosing suicidal thoughts. Greater efforts to reduce structural barriers, destigmatize mental illness, and provide culturally competent information about mental health and suicide are needed.

Results also indicated that those who were never married were more likely than those who are currently married to disclose suicidal ideation. Prior research has demonstrated that being married is protective of suicide risk relative to being unmarried or divorced/separated (Øien‐Ødegaard et al., 2021). It is important to note that we are unsure whether participants who endorsed suicidal ideation and disclosures were married at the time of these experiences. Those who are experiencing suicidal thoughts and are married are not necessarily representative of most married individuals. However, there is some research to suggest that, among those who are married, marital discord is associated with suicidal thoughts and behaviors (Robustelli et al., 2015). In light of these findings, it is possible that those who are currently married disclose ideation less, either because they are no longer experiencing suicidal ideation or because they are experiencing difficulties in their marriage and are less willing to discuss their suicidal thoughts with their partner. Those experiencing marital discord may feel more isolated than those who are never married, resulting in decreased likelihood of disclosure. Ultimately, much more research is needed to determine the replicability of this finding and to parse apart the potential reasons for disclosure to be less likely among currently married individuals.

Results from the present study did not indicate an association between age or sex and disclosing suicidal thoughts. As previously discussed, prior research on the association between sociodemographics and likelihood of disclosing suicidal thoughts has been mixed. The present study generally suggests that age and sex are likely not the most decisive factor for understanding to whom adults are more likely to disclose.

Consistent with prior research, those who had made a prior suicide attempt were more likely to disclose their suicidal thoughts than those who had never attempted suicide (Calear & Batterham, 2019). Attempting suicide is harder to conceal than having suicidal thoughts; therefore, this finding may in part be related to the increased difficulty in keeping suicidal behaviors private. Additionally, while some people may be dismissive of their suicidal thoughts, it is harder to be dismissive of suicide attempts. For some, escalation may indicate that they need help and lead them to reach out and disclose their suicidal thoughts to others. It is encouraging to see individuals with prior attempts disclosing their suicidal thoughts given their heightened suicide risk; however, the majority of those who die by suicide do so on their first attempt (Brent et al., 1993; Shaffer et al., 1996). Those who are experiencing suicidal thoughts without an attempt are therefore at an important point for intervention. Suicidal ideation is a primary risk of morbidity, contributes to prolonged suffering, and should be a primary target of intervention (Jobes & Joiner, 2019). Efforts to dispel myths about what happens when someone discloses suicidal ideation are needed to increase rates of disclosure. Further efforts to educate and provide resources are needed to increase understanding of the importance of disclosing these experiences.

Results also indicated that those with prior mental health treatment and those with more positive attitudes about seeking mental health treatment were more likely to have disclosed their suicidal thoughts. More positive attitudes toward seeking treatment have been previously associated with a greater likelihood of mental health treatment (ten Have et al., 2010). Indeed, attitudes toward help seeking and prior mental health treatment were positively correlated within our sample. Engaging with mental health services allows for opportunities to disclose to a professional, an opportunity unavailable to those who have not engaged with services. Additionally, those who have sought mental health treatment may have also been exposed to more psychoeducation about the importance of asking for help and disclosing suicidal thoughts relative to those who have never been in treatment. These individuals may not just have an increased opportunity to disclose to professionals but also may be more open to disclose these experiences to personal connections. These findings highlight the importance of reaching those who might have more negative attitudes toward seeking mental health treatment.

Given the heightened suicide risk that arises when one experiences suicidal thoughts and has access to a firearm, finding that firearm owners are not less likely to disclose suicidal thoughts is encouraging. Prior work has found that firearm owners were less likely to endorse suicidal ideation on some assessments, particularly as their risk for suicide increases (Bryan et al., 2022). Further, firearm‐owning service members who do not disclose their suicidal ideation were more likely to store firearms unsecured (Anestis et al., 2023). As such, direct disclosures offer an important indicator of risk that could be the point at which secure firearm storage is addressed. Given current findings that disclosure to personal connections are more likely than to professionals, efforts to equip the public with information about the association between firearms and suicide along with secure storage options would aid in individuals' abilities to respond to suicide disclosures by firearm owners.

We also sought to examine the extent to which different disclosure sources were perceived as helpful. Consistent with expectations, professionals were perceived to be more helpful relative to personal connections. The mean helpfulness rating for mental health professionals was 4.01, where 4 was considered “Somewhat helpful.” The mean helpfulness rating for personal connections was 3.74, where 3 was considered to be “Neither harmful nor helpful.” It is likely that professionals are better equipped to understand, react, and provide resources for those experiencing suicidal thoughts. Personal connections on the other hand likely lack some of this knowledge and the added complication of interpersonal relationships may create a situation where these disclosures do not prove to be as beneficial. When participants rated both personal and professional connections, perceived helpfulness was statistically equal. This finding suggests that, when given access to both sources, those experiencing suicidal ideation can benefit equally from disclosing. However, only about a quarter of those who disclosed used both personal and professional sources, leaving almost 75% of those who disclose to only tell one or the other. Given that disclosures to personal connections were nearly two times more common than those to professionals, programs for the public providing psychoeducation and resources for dealing with suicidal thoughts and behaviors are necessary, especially to mitigate the risk of reacting in a way that is perceived as harmful.

As anticipated and consistent with prior literature, disclosures to personal connections were more frequent than disclosures to professionals. Not all individuals who experience suicidal thoughts seek mental health treatment; therefore, many people with suicidal thoughts lack the opportunity to disclose them to a professional. For example, prior research found that more than half of young adults who reported suicidal thoughts in the year prior did not engage with mental health treatment in that year (Substance Abuse and Mental Health Services Administration [SAHMSA], 2016). It is probable that personal disclosures are more prevalent as they are more available. Another consideration is that individuals in mental health treatment may not be comfortable disclosing their thoughts of suicide to mental health providers (Blanchard & Farber, 2016). Determining ways to encourage mental health treatment for those with suicidal thoughts and ensuring they feel comfortable disclosing this information to providers is necessary. Finally, given that individuals more often disclose to personal connections, it is also important to ensure such disclosures are helpful.

Among personal disclosures, results indicated that parents (*M*_rating_ = 3.52) were rated as least helpful. While the mean differences are small, it is also important to note that the greatest variance in harmful/helpful ratings was for parents (SD = 1.25), suggesting that there is more variability in how parents respond to suicidal ideation disclosures. As such, some participants experienced harmful disclosures which can dissuade future disclosures. This could leave those who disclose without the help they were seeking when revealing their thoughts of suicide, emphasizing the importance of psychoeducation for the general public. Efforts to educate parents about the prevalence of suicidal thoughts and behaviors as well as to provide resources which parents can use in case their adult child reports having these experiences are necessary.

Among professionals, results indicated that psychologists were rated most helpful (*M*_rating_ = 4.31), while internet therapy programs were least helpful (*M*_rating_ = 3.59). Helpfulness ratings of psychologists are somewhat surprising as a growing body of literature indicates that professional suicide assessment and determination of suicide risk are generally unhelpful in preventing suicide (Rudd, 2023). However, it seems that in response to a direct disclosure, psychologists may be offering empathy and resources that are perceived as helpful. Internet therapy programs were rated as the least helpful; this was also the least utilized professional source. Our study did not provide specific guidelines for what an “internet therapy program” is, so it is unclear whether the 10 participants who endorsed utilizing this source were picturing a self‐help program or a practitioner who offers online therapy. Online self‐help options have been shown to be helpful for reducing suicidal thoughts (e.g., De Jaegere et al., 2019); however, in the current study other professional sources were rated as more helpful than the online source. It is possible that disclosing to an app, bot, or other through a computer feels less personal and, as such, less helpful. Future research should work to discern differences between typical professional disclosures (e.g., in person or to a provider directly) from online program disclosures.

This study is not without limitations. We relied on retrospective, cross‐sectional self‐reports, introducing the possibility that participants' circumstances may have changed over time and may not be the same now as when they were experiencing suicidal ideation and/or disclosing. Some individuals may have disclosed many years ago whereas other may have done so more recently. Time and space from these disclosures may shape how individuals perceive the events or allow individuals to forget their disclosure completely. Research assessing people's immediate reactions to disclosing is needed. Similarly, a limitation to our data is the focus on lifetime ideation rather than more recent reports which introduces potential bias and inconsistent reporting (Klimes‐Dougan et al., 2022). Future research should employ longitudinal methods and adding in more specific inquiries of time‐periods. We did not examine reasons for perceived helpful/harmful ratings. Qualitative research aimed to explore reasons may be useful for tailoring public health efforts for personal disclosures and professional trainings for disclosures to providers. The content and context of the disclosure were not examined. Individuals may have disclosed ideation passively (e.g., telling someone that they wish to sleep and never wake up). Sources may then perceive the ideation as less severe and react in a minimal way that is perceived as less helpful. On the other hand, individuals with passive ideation may not perceive their thoughts as a form of suicidal ideation or severe enough to warrant support, and they may not disclose their thoughts at all. Future work should consider differentiating between passive and active ideation, as well as inquiring about the context of disclosures. Finally, some of the present study's measures were limited. We utilized PTCI items as a proxy of mistrust, which has not been validated. While internal consistency for these isolated items was good in the current investigation and extant literature (e.g., Anestis & Bryan, 2021), further research with validated measures of mistrust is needed. We also did not assess for gender or have more nuanced assessment of racial and ethnic identity which limits our ability to understand these identities as they relate to disclosures.

Overall, the present study adds to a growing body of literature examining disclosures of suicidal thoughts. Several demographic and clinical characteristics were associated with likelihood of prior disclosure and these results offer an avenue for future programs to increase disclosure among those who are less likely to do so. Our results suggest that, overall, these disclosures are not typically perceived as harmful; however, for many individuals they are also not perceived as helpful. These findings suggest that programs that provide education and resources for suicide prevention are needed so that disclosures to personal connections can be effective. From the professional perspective, further work toward training various providers on how to assess and address suicide risk effectively is needed. Implementation of such programs may aid in suicide prevention efforts.

## CONFLICT OF INTEREST STATEMENT

The authors do not have any conflicts of interest to disclose.

## Data Availability

Data can be made available upon responsible request to the corresponding author.

## References

[sltb13126-bib-0002] Anestis, M. D. , Bond, A. E. , Capron, C. , Bryan, A. O. , & Bryan, C. J. (2023). Differences in firearm storage practices among United States military servicemembers who have and have not disclosed suicidal thoughts or attended behavioral health sessions. Suicide & Life‐Threatening Behavior, 53(2), 262–269. 10.1111/sltb.12940 36622136

[sltb13126-bib-0043] Anestis, M. D. , & Bryan, C. J. (2021). Threat perceptions and the intention to acquire firearms. Journal of Psychiatric Research, 133, 113–118. 10.1016/j.jpsychires.2020.12.033 33338733

[sltb13126-bib-0003] Blanchard, M. , & Farber, B. A. (2016). Lying in psychotherapy: Why and what clients don't tell their therapist about therapy and their relationship. Counselling Psychology Quarterly, 29(1), 90–112. 10.1080/09515070.2015.1085365

[sltb13126-bib-0004] Bommersbach, T. J. , Rosenheck, R. A. , & Rhee, T. G. (2023). Racial and ethnic differences in suicidal behavior and mental health service use among US adults, 2009–2020. Psychological Medicine, 53(12), 5592–5602. 10.1017/S003329172200280X 36106374 PMC10482716

[sltb13126-bib-0005] Bond, A. E. , Bandel, S. L. , Rodriguez, T. R. , Anestis, J. C. , & Anestis, M. D. (2022). Mental health treatment seeking and history of suicidal thoughts among suicide decedents by mechanism, 2003‐2018. JAMA Network Open, 5(3), e222101. 10.1001/jamanetworkopen.2022.2101 35285919 PMC9907334

[sltb13126-bib-0006] Brent, D. A. , Perper, J. A. , Moritz, G. , Allman, C. , Friend, A. , Roth, C. , Schweers, J. , Balach, L. , & Baugher, M. (1993). Psychiatric risk factors for adolescent suicide: A case‐control study. Journal of the American Academy of Child and Adolescent Psychiatry, 32(3), 521–529. 10.1097/00004583-199305000-00006 8496115

[sltb13126-bib-0007] Bryan, C. J. , Bryan, A. O. , Wastler, H. M. , Khazem, L. R. , Ammendola, E. , Baker, J. C. , Szeto, E. , Tabares, J. , & Bauder, C. R. (2022). Assessment of latent subgroups with suicidal ideation and suicidal behavior among gun owners and non‐gun owners in the US. JAMA Network Open, 5(5), e2211510. 10.1001/jamanetworkopen.2022.11510 35544138 PMC9096594

[sltb13126-bib-0008] Calear, A. L. , & Batterham, P. J. (2019). Suicidal ideation disclosure: Patterns, correlates and outcome. Psychiatry Research, 278, 1–6. 10.1016/j.psychres.2019.05.024 31128420

[sltb13126-bib-0009] Chu, J. P. , Hsieh, K. Y. , & Tokars, D. A. (2011). Help‐seeking tendencies in Asian Americans with suicidal ideation and attempts. Asian American Journal of Psychology, 2(1), 25–38. 10.1037/a0023326

[sltb13126-bib-0010] Cohen, J. (1977). Statistical power analysis for the behavioral sciences. Academic Press.

[sltb13126-bib-0011] Conner, A. , Azrael, D. , & Miller, M. (2019). Suicide case‐fatality rates in the United States, 2007 to 2014. Annals of Internal Medicine, 171(12), 885–895. 10.7326/M19-1324 31791066

[sltb13126-bib-0012] Cukrowicz, K. C. , Duberstein, P. R. , Vannoy, S. D. , Lin, E. H. , & Unützer, J. (2014). What factors determine disclosure of suicide ideation in adults 60 and older to a treatment provider? Suicide & Life‐Threatening Behavior, 44(3), 331–337. 10.1111/sltb.12075 24494695

[sltb13126-bib-0013] Curtin, S. C. , Garnett, M. F. , & Ahmad, F. B. (2023). Provisional estimates of suicide by demographic characteristics: United States, 2022. Vital Statistics Rapid Release, 34, 1–9. 10.15620/cdc:133702

[sltb13126-bib-0014] De Jaegere, E. , van Landschoot, R. , van Heeringen, K. , van Spijker, B. A. J. , Kerkhof, A. J. F. M. , Mokkenstorm, J. K. , & Portzky, G. (2019). The online treatment of suicidal ideation: A randomised controlled trial of an unguided web‐based intervention. Behaviour Research and Therapy, 119, 103406. 10.1016/j.brat.2019.05.003 31176889

[sltb13126-bib-0015] DeFreitas, S. C. , Crone, T. , DeLeon, M. , & Ajayi, A. (2018). Perceived and personal mental health stigma in Latino and African American college students. Frontiers in Public Health, 6, 49. 10.3389/fpubh.2018.00049 29536000 PMC5834514

[sltb13126-bib-0016] Elhai, J. D. , Schweinle, W. , & Anderson, S. M. (2008). Reliability and validity of the Attitudes Toward Seeking Professional Psychological Help Scale‐Short Form. Psychiatry Research, 159(3), 320–329. 10.1016/j.psychres.2007.04.020 18433879

[sltb13126-bib-0042] Encrenaz, G. , Kovess‐Masféty, V. , Gilbert, F. , Galéra, C. , Lagarde, E. , Mishara, B. , & Messiah, A. (2012). Lifetime risk of suicidal behaviors and communication to a health professional about suicidal ideation. Results from a large survey of the French adult population. Crisis, 33(3), 127–136. 10.1027/0227-5910/a000113 22450035

[sltb13126-bib-0039] Eskin, M. (2003). A cross‐cultural investigation of the communication of suicidal intent in Swedish and Turkish adolescents. Scandinavian Journal of Psychology, 44(1), 1–6. 10.1111/1467-9450.t01-1-00314 12602997

[sltb13126-bib-0017] Fischer, E. H. , & Farina, A. (1995). Attitudes toward seeking professional psychological help: A shortened form and considerations for research. Journal of College Student Development, 36, 368–373.

[sltb13126-bib-0018] Foa, E. B. , Ehlers, A. , Clark, D. M. , Tolin, D. F. , & Orsillo, S. M. (1999). The Posttraumatic Cognitions Inventory (PTCI): Development and validation. Psychological Assessment, 11(3), 303–314.

[sltb13126-bib-0019] Fulginiti, A. , Pahwa, R. , Frey, L. M. , Rice, E. , & Brekke, J. S. (2016). What factors influence the decision to share suicidal thoughts? A multilevel social network analysis of disclosure among individuals with serious mental illness. Suicide and Life‐threatening Behavior, 46(4), 398–412. 10.1111/sltb.12224 26511676

[sltb13126-bib-0020] Garnett, M. F. , & Curtin, S. C. (2023). Suicide mortality in the United States, 2001–2021. NCHS data brief, no 464. National Center for Health Statistics. 10.15620/cdc:125705 37093258

[sltb13126-bib-0021] Hallford, D. J. , Rusanov, D. , Winestone, B. , Kaplan, R. , Fuller‐Tyszkiewicz, M. , & Melvin, G. (2023). Disclosure of suicidal ideation and behaviours: A systematic review and meta‐analysis of prevalence. Clinical Psychology Review, 101, 102272. 10.1016/j.cpr.2023.102272 37001469

[sltb13126-bib-0022] Husky, M. M. , Zablith, I. , Alvarez, F. V. , & Kovess‐Masfety, V. (2016). Factors associated with suicidal ideation disclosure: Results from a large population‐based study. Journal of Affective Disorders, 205, 36–43. 10.1016/j.jad.2016.06.054 27400193

[sltb13126-bib-0023] Jobes, D. A. , & Joiner, T. E. (2019). Reflections on suicidal ideation. Journal of Crisis Intervention and Suicide, 40(4), 227–230. 10.1027/0227-5910/a000615 31274031

[sltb13126-bib-0024] Kim, J. H. (2019). Multicollinearity and misleading statistical results. Korean Journal of Anesthesiology, 72(6), 558–569. 10.4097/kja.19087 31304696 PMC6900425

[sltb13126-bib-0025] Klimes‐Dougan, B. , Mirza, S. A. , Babkin, E. , & Lanning, C. (2022). Biased reporting of past self‐injurious thoughts and behaviors: A literature review. Journal of Affective Disorders, 308(1), 596–606. 10.1016/j.jad.2022.04.073 35429538

[sltb13126-bib-0027] Nock, M. K. , Holmberg, E. B. , Photos, V. I. , & Michel, B. D. (2007). The self‐injurious thoughts and behaviors interview: Development, reliability, and validity in an adolescent sample. Psychological Assessment, 19, 309–317. 17845122 10.1037/1040-3590.19.3.309

[sltb13126-bib-0028] Øien‐Ødegaard, C. , Hauge, L. J. , & Reneflot, A. (2021). Marital status, educational attainment, and suicide risk: A Norwegian register‐based population study. Population Health Metrics, 19, 33. 10.1186/s12963-021-00263-2 34247635 PMC8273935

[sltb13126-bib-0029] Osvath, P. , Michel, K. , & Fekete, S. (2003). Contacts of suicide attempters with healthcare services in pecs and Bern in the WHO/EURO multicentre study on Parasuicide. International Journal of Psychiatry in Clinical Practice, 7(1), 3–8. 10.1080/13651500310001004 24937235

[sltb13126-bib-0030] Robustelli, B. L. , Trytko, A. C. , Li, A. , & Whisman, M. A. (2015). Marital discord and suicidal outcomes in a National Sample of married individuals. Suicide & Life‐Threatening Behavior, 45(5), 623–632. 10.1111/sltb.12157 25752755

[sltb13126-bib-0031] Rudd, M. D. (2023). Recognizing flawed assumptions in suicide risk assessment research and clinical practice. Psychological Medicine, 53(5), 2186–2187. 10.1017/S0033291721002750 34229771 PMC10106284

[sltb13126-bib-0032] Shaffer, D. , Gould, M. S. , Fisher, P. , Trautman, P. , Moreau, D. , Kleinman, M. , & Flory, M. (1996). Psychiatric diagnosis in child and adolescent suicide. Archives of General Psychiatry, 53(4), 339–348. 10.1001/archpsyc.1996.01830040075012 8634012

[sltb13126-bib-0040] Shin, K. E. , Spears, A. P. , Zhang, R. , & Cha, C. B. (2023). Suicide‐related disclosure patterns among culturally minoritized youth: Examining differences across race, ethnicity, gender identity, and sexual orientation. Suicide & Life‐Threatening Behavior, Advance online publication. 10.1111/sltb.13026 PMC13542463 38032047

[sltb13126-bib-0033] Stone, D. M. , Jones, C. M. , & Mack, K. A. (2021). Changes in suicide rates—United States, 2018‐2019. MMWR. Morbidity and Mortality Weekly Report, 70(8), 261–268. 10.15585/mmwr.mm7008a1 33630824 PMC8344989

[sltb13126-bib-0034] Substance Abuse and Mental Health Services Administration . (2016). Suicidal thoughts and behavior among adults: Results from the 2015 National Survey on Drug Use and health. https://www.samhsa.gov/data/sites/default/files/NSDUH‐DR‐FFR3‐2015/NSDUH‐DR‐FFR3‐2015.htm#:~:text=Among%20adults%20in%202015%20who,but%20did%20not%20obtain%20care 29792622

[sltb13126-bib-0035] ten Have, M. , de Graaf, R. , Ormel, J. , Vilagut, G. , Kovess, V. , Alonso, J. , & ESEMeD/MHEDEA 2000 Investigators . (2010). Are attitudes towards mental health help‐seeking associated with service use? Results from the European study of epidemiology of mental disorders. Social Psychiatry and Psychiatric Epidemiology, 45(2), 153–163. 10.1007/s00127-009-0050-4 19381427 PMC2820660

[sltb13126-bib-0036] U.S. Surgeon General . (2021). The surgeon general's call to action to implement the national strategy for suicide prevention. https://www.hhs.gov/sites/default/files/sprc‐call‐to‐action.pdf 37347878

[sltb13126-bib-0037] Uebelacker, L. A. , Marootian, B. A. , Pirraglia, P. A. , Primack, J. , Tigue, P. M. , Haggarty, R. , Velazquez, L. , Bowdoin, J. J. , Kalibatseva, Z. , & Miller, I. W. (2012). Barriers and facilitators of treatment for depression in a latino community: A focus group study. Community Mental Health Journal, 48(1), 114–126. 10.1007/s10597-011-9388-7 21267653

[sltb13126-bib-0038] Ward, E. C. , Wiltshire, J. C. , Detry, M. A. , & Brown, R. L. (2013). African American men and women's attitude toward mental illness, perceptions of stigma, and preferred coping behaviors. Nursing Research, 62(3), 185–194. 10.1097/NNR.0b013e31827bf533 23328705 PMC4279858

